# Mr. Paget’s Report Continued

**Published:** 1844-04-06

**Authors:** 


### MR. PAGET'S REPORT CONTINUED.

#### TRANSFORMATIONS OF NUTRITIOUS SUBSTANCES.

Among the numerous papers written on the transformations which the food undergoes in its passage in the body, the most interesting and almost the only ones which afford any definite conclusions are those relating to the formation of fatty matters from the saccharine and starchy principles. It seemed to be proved by Huber's experiments on bees that wax could be formed by them out of pure sugar or honey; for when their food contained nothing but one of these they formed their combs as usual. M. Dumas, who had opposed Liebig's deductions from these facts, suspected that the wax might be formed from the fat which the bees had in their own bodies before they commenced their purely saccharine diet. He therefore, with M. Milne Edwards, repeated the experiment, and in a successful trial obtained the following results: 1988 bees were inclosed in a hive, and from an analysis of the bodies of 117 from the same stock it was estimated that the bodies of the 1988 contained 3·218 grammes of fatty matter. The honey on which they fed contained 8-10,000th of waxy matter. The experiment was continued thirty-one days, and the bees consumed 834·889 grammes of honey, and produced 11·515 grammes of wax, or at the rate of 0·0064 for each bee. After the experiment 105 bees were analysed, and yielded 0·442 gr. of fatty matter, or at the rate of 0·0042 gr. each. Thus the fatty matter pre-existing in each bee was 0·0018 gr., and the quantity furnished to each in its food was 0·0038: but each produced in thirty-one days 0·0064 gr., and each at the end contained 0·0042, giving a total of fatty matter 0·0106, and an excess, which must have been formed by transformation of the food, equal to 0·00742 gr. per bee.

The old view of the production of the oleaginous constituents of the bodies of herbivora by the transformation of the saccharine and amylaceous principles of their food is thus confirmed; and the evidence is the better for its being honestly published by one who had been the chief opponent of the view. Connected with it is a fact recently observed by MM. Pelouze and Gelis, that under certain circumstances, butyric acid is formed during the fermentation of sugar. By the action of the acid thus obtained upon glycerin they formed also butyrin, another of the constituents of butter. Still, however, the results of the experiments on which Dumas' former opinion was founded are important, as proving that the several articles of food of the herbivora contain a much larger proportion of fatty matter than had been imagined. In maize and other grains, for example, he has found from 7 to 9 percent., and in grass, hay, &c. considerable proportions, which in all probability contribute to the formation of the fat, though they are not its only source.

*Assimilation.* In the last Report some remarkable observations were referred to, proving the analogies between the forms assumed by certain inorganic precipitates such as those of the carbonates of lime and iron, and the forms of the nuclei and cells of organic tissues. Now, Dr. Hermann Jordan, of Saarbrück, has called attention to the phenomena of the reparation of damaged crystals, as bearing analogy to the repair of injured organized bodies. The facts which he establishes are these: 1. Any portion of crystal —whatever surfaces, angles, or edges may have been removed from it—may, under proper circumstances, repair itself into a complete individual; that is, restore itself to the same form which it would have had if no injury had been done to it. 2. At the same time with the reproduction of the truncated part, a growth of the whole crystal takes place: *but the effort of the formative act is especially directed to replace the lost part.* 3. The effort at reparation stands in a direct relation to the extent of the loss, and decreases in proportion as the loss is replaced. 4. He points out the mode in which the process of reparation takes place, and concludes that his examinations "have demonstrated the tendency of individuals to maintain their integrity and to replace material losses by the formation of more matter, according to the type of their original form, as a phenomenon to be observed as well in inorganic as in organic nature—as a phenomenon which belongs to the individual as such, whether it have a membered body or the simple structure of a crystal."

ORGANS OF ANIMAL LIFE.

*Chemical composition of bone.* The following are the results of the most recent and careful analyses of human bone, by Marchand and Lehmann. In both cases the bones were deprived of fat and periosteum: in each the average of six examinations is given; Marchand's were made on thigh-bones, Lehmann's on the long bones of the arm and leg.

| | | | Marchand. | Lehmann. |
|---|---|---|---|---|
| Organic mat. | Cartilage insol. in h.cl. | 27.23 | 33.26 | 32.56 |
| | " soluble " | 5·02 | | |
| | Vessels | 1.01 | | |
| Phosphate of lime | | | 52·26 | 54·61 |
| Fluate of lime | | | 1· | |
| Carbonate of lime | | | 10·21 | 9·41 |
| Phosphate of magnesia | | | 1·05 | 1·07 |
| Soda | | | 0·92 | 1·11 |
| Hydrochlorate of Soda | | | 0·25 | 0·38 |
| Oxydes of iron and manganese and loss | | | 1.05 | ·86 |
| | | | 100· | 100· |

The following are average relative proportions of organic and earthy matter, collected by Lehmann from his own and the analyses of two other observers.

| | Sebastian. | Lehmann. | Frerichs. Compact bone. | Frerichs. Spongy bone, |
|---|---|---|---|---|
| Organic | 36·66 | 32·28 | 31·2 | 37·82 |
| Earthy | 63·34 | 67·72 | 68·8 | 62·18 |

All found that the earthy matter increases with age.

In a memoir on ancient and fossil bones, M. Gerardin states that the degrees of alteration which buried bones undergo depend almost entirely on the degrees in which the soils are exposed to air and moisture. They always lose more or less of their animal matter; and sometimes, when they lie in a soil traversed by streams of water, it is completely removed: the ammonia proceeding from the part first decomposed saponifies the rest and makes it soluble. In human bones long buried and in fossil bones there is always more subphosphate of lime than in recent bones; in *human bones* long buried the proportion of carbonate of lime is generally diminished, in fossil bones it is increased. In fossil bones also there is always some fluate of lime; in human bones, under whatever circumstances, there is none: it seems to be introduced into fossil bones by infiltration from without, and its presence may be depended on as a sign that a bone is really fossilized.

*Structure of bone.* Dr. Fleischmann has described the minute structure of *vegetable ivory*, from the fruit of the *manicaria saccifera*, (Gärtner,) a species of palm growing near the coast of Guiana, as being closely analogous to that of bone, at least in regard to the *corpuscles* which it presents. It possesses also, he says, somewhat of the chemical properties of bone. Thin sections exhibit the most beautiful structures, like the bone-corpuscles, except that they are more regular, and lie within regularly-formed cells, of which they appear to be the nuclei. Branches like the calcigerous canals proceed from each corpuscle, but do not give off smaller branches nor extend beyond the wall of the cell; each branch ends within the cell-wall in a bluntly-closed extremity. He believes that there is the same arrangement in true bone; that each corpuscle has, like a nucleus, a distinct cell-wall around it, such as he has figured in a section of bone from a child; that the canals of the corpuscles are unbranched, and that they end within the cell-walls, having only an appearance of anastomosis with the canals in adjacent cells.

The chapter on 'Bone,' in the 'Physiological Anatomy' of Todd and Bowman, contains by far the best plates yet published of the minute osseous structure. The ultimate structure also is there described, from preparations made by Mr. Tomes, to be granular. The ultimate granules vary in size from 1-6000th to 1-14000th of an inch; they are oval or oblong, and cohere firmly, possibly by the medium of some second substance. In some instances Mr. Tomes has met with a very minute network, which seems adapted to receive them in its interstices; but this he considers, requires confirmation.

*Process of ossification.* In the same work is a description of the process of ossification, which is, in several points, new and interesting, (p. 117.) In the vicinity of the point of ossification the nucleated cartilage-cells (which usually are scattered irregularly) arrange themselves in linear series, which run down, as it were, to the ossifying surface. At first the series are small and not regular, but nearer to the ossifying part they form rows of twenty or thirty. The cells in these rows are closely compressed, and their nuclei seem flattened. The lowest rows dip into and rest in deep narrow cups of bone, formed by the osseous transformation of the intercellular substance between the rows, and as ossification advances these cups are converted into close areolæ or *cancelli*, with extremely thin lamelliform walls. Immediately upon the ossifying surface, nuclei, which before were closely compacted, separate considerably from one another by the increase of material within the cells: they also often enlarge and become more transparent. Deeper in the new bone the lamellæ which inclose the cancelli, and which were formed by the ossification of the intercellular substance, are found thicker and more like perfect bone: they include in their substance elongated oval spaces, which, except that they are roughly granular, exactly resemble the ordinary *bone-corpuscles*, and which are evidently the nuclei of the cells of the temporary cartilages. The curvilinear outline of the now ossified cells of these nuclei can often be discerned. Within the cancelli only a few cells can be detected, these cavities (of the cancelli) being chiefly occupied by a quantity of new substance, consisting of granules, and resembling a formative blastema or basis. It thus appears that after the ossification of the intercellular substance, (by which are formed the lamellæ which are the walls of the cancelli,) the rows of cartilage all arrange themselves on the inner surface of these newly-formed cancelli, and are ossified, with the exception of their nuclei, which remain granular, and subsequently form the corpuscles of bone; and that a new substance or blastema appears within the cancelli, from which, probably, vessels are developed, and the further steps in the growth of the bone proceed.

